# TGF-β signaling can act from multiple tissues to regulate *C. elegans* body size

**DOI:** 10.1186/s12861-014-0043-8

**Published:** 2014-12-06

**Authors:** Aidan Dineen, Jeb Gaudet

**Affiliations:** Department of Biochemistry and Molecular Biology, Alberta Children’s Hospital Research Institute, University of Calgary, Calgary, T2N 4 N1 Alberta Canada

**Keywords:** *Caenorhabditis elegans*, TGF-β, Sma/Mab, Body size, Pharynx, Hypodermis

## Abstract

**Background:**

Regulation of organ and body size is a fundamental biological phenomenon, requiring tight coordination between multiple tissues to ensure accurate proportional growth. In *C. elegans*, a TGF-β pathway is the major regulator of body size and also plays a role in the development of the male tail, and is thus referred to as the TGF-β/Sma/Mab (for *sma*ll and *m*ale *ab*normal) pathway. Mutations in components of this pathway result in decreased growth of animals during larval stages, with Sma mutant adults of the core pathway as small as ~60-70% the length of normal animals. The currently accepted model suggests that TGF-β/Sma/Mab pathway signaling in the *C. elegans* hypodermis is both necessary and sufficient to control body length. However, components of this signaling pathway are expressed in other organs, such as the intestine and pharynx, raising the question of what the function of the pathway is in these organs.

**Results:**

Here we show that TGF-β/Sma/Mab signaling is required for the normal growth of the pharynx. We further extend the current model and show that the TGF-β/Sma/Mab pathway can function in multiple tissues to regulate body and organ length. Specifically, we find that pharyngeal expression of the SMAD protein SMA-3 partially rescues both pharynx length and body length of *sma-3* mutants.

**Conclusions:**

Overall, our results support a model in which the TGF-β/Sma/Mab signaling pathway can act in multiple tissues, activating one or more downstream secreted signals that act non cell-autonomously to regulate overall body length in *C. elegans*.

**Electronic supplementary material:**

The online version of this article (doi:10.1186/s12861-014-0043-8) contains supplementary material, which is available to authorized users.

## Background

An important question in developmental biology is what controls growth at three levels: the organism, the organ and the individual cells [1]. Organismal size appears to be regulated by multiple inputs including genetic pathways that are active during development to regulate cell number (cell proliferation and apoptosis) and cell size. Overall body size of an organism also responds to environmental cues such as nutrient availability and stress. Many of these environmental inputs converge on the Tor signaling pathway, which regulates multiple downstream targets to ultimately control both cell size and cell division [2]. Similarly, the size of an organ can be determined by cell number and/or cell size, again controlled by genetic and environmental components. In *Drosophila* and mammals, the regulation of individual cell size is controlled in part by a conserved insulin signaling pathway that receives nutritional input and translates this information to regulate cellular metabolism [3].

Growth of the nematode *C. elegans* occurs through both increase in cell number and increase in cell size. From hatching to adulthood, the number of somatic nuclei increases from 550 to 959 [4], while cells also increase in size. As in other animals, *C. elegans* body size is regulated by nutrient status. For example, animals defective in feeding are significantly smaller than wild type animals [5,6]. Genetic regulation of size involves at least two signaling pathways: a much less studied pathway that includes the MAP kinase SMA-5, and the major pathway involving TGF-β [7,8]. These two pathways act non-redundantly in body size regulation and may also act independently of nutritional status [9,10]. Additionally, body size can be constrained by morphology defects in the extracellular cuticle surrounding the worm resulting in the Dpy phenotype [11,12].

The TGF-β pathway is referred to as the Sma/Mab pathway because loss of function mutations lead to both small body length (Sma phenotype) and male tail defects (Mab phenotype). The pathway ligand, DBL-1, is expressed in a set of neurons, including some pharyngeal neurons [13,14]. Binding of DBL-1 to the Type I/II receptors SMA-6 and DAF-4 activates the downstream effector SMADs SMA-2, −3 and −4, which function together with the Schnurri homolog SMA-9 to regulate transcription of target genes in *C. elegans*, none of which have yet been identified [15]. Previous work has suggested that TGF-β/Sma/Mab signaling acts solely in the hypodermis to control organismal length [16,17]. However, components of the pathway, such as SMA-3 and SMA-6, are expressed in additional tissues, namely the pharynx and intestine [16,17], where it has been suggested that they regulate innate immunity genes [10,18,19].

We have previously reported that the pharynges of *sma-2(e502)* and *sma-3(e491)* mutants are shorter in length than wild type [20]. To test the hypothesis that pharynx length is regulated by TGF-β/Sma/Mab signaling in pharyngeal cells, we performed rescue experiments in which *sma-3(+)* was expressed under the control of different tissue-specific promoters. We found that expression of *sma-3* in the pharynx could partially rescue both pharynx length and body length of *sma-3* mutants, in contrast to expectation based on the prevailing model. Our findings suggest that TGF-β/Sma/Mab signaling can function in multiple tissues (hypodermis and pharynx) to control organ and overall body length.

## Methods

### *C. elegans* strains

Standard nematode handling conditions were used [11]. Animals were grown at 20°C. Strains used were wild type N2, CB61 *dpy-5(e61) I,* DR1785 *mIn1[dpy-10(e128)]/unc-4(e120) II*, CB1482 *sma-6(e1482) II,* CB491 *sma-3(e491) III*, CS24 *sma-3(wk30) III* (kindly provided by Dr. Cathy Savage-Dunn, Queens College), MT468 *dpy-7(e88) unc-6(n102) X* and JM228 ctIs40[*dbl-1(+) sur-5::gfp*] *X; sma-3(e491) III; ivEx163[myo-2p::sma-3 marg-1p::sma-3 phat-1p::yfp elt-2p::tdTomato::His2B]*.

### Plasmid construction

To construct a *sma-3* minigene, we amplified *sma-3* cDNA from a library using primers oGD861 acggtaccATGAACGGATTACTGCATATGCATGGTC and oGD860 tagagctcTTATGTCATTGAATTTGGTTCCATCAAGTTCG; for all oligos, uppercase sequence corresponds to gene sequence; lowercase corresponds to restriction site-containing sequence or plasmid sequence that facilitates cloning. This 1.2 kb fragment was cut with *Kpn*I and *Sac*I and cloned into the *myo-2*-containing plasmid pSEM474 [21] to create a *myo-2p::sma-3(cDNA)* plasmid. We next amplified a 2.9 kb genomic *sma-3* fragment from N2 DNA using the same oligos and digested with *Bgl*II and *Sal*I to isolate a 1 kb genomic fragment containing exons 2–8 (and introns 2–7). This genomic fragment was cloned into the *myo-2p::sma-3(cDNA)* clone to generate *myo-2p::sma-3* minigene. The *sma-3* minigene was sequenced to ensure that no mutations were introduced during cloning.

Other minigene constructs contained the same *sma-3* minigene cassette but with different promoter sequences, amplified with the following pairs of primers from either genomic N2 DNA or pRF4 (in the case of *rol-6*):

*sma-3:* oGD3 gctgaaatcactcacaacgatgg

oGD1230 cggggtaccTTGCTCTCATTTCAAAAAAACTAATTC

*marg-1*: oGD316 aactgcagATCAAAGTGCCGATCGAAGT

oGD317 ggggtaccGTTGGAGGAGCCATTGAGA

*rol-6*: oGD1047 gagactgcagGTTTTGATAAAATTGTGGTGTAGTCCATAATG

oGD1048 gagaggtaccCTGGAAATTTTCAGTTAGATCTAAAGATATATCC

The K07C11.4 promoter was cloned from the previously described reporter plasmid pSEM900 [22].

To examine *sma-3* expression, we amplified the entire *sma-3* gene, including ~1.2 kb of sequence upstream of the predicted ATG (the entire intergenic region), using the primers oGD956 (caactgcagCTTGCTAACTGTGTCCCCAACCATC) and oGD957 (catggtaccGTCATTGAATTTGGTTCCATCAAGTTCG). We digested this fragment with *Pst*I and *Kpn*I and cloned it into the GFP expression vector pPD95.77, creating an in-frame translational fusion between *sma-3* and *gfp*. To create a *sma-3p::sma-3* minigene, we isolated a *Pst*I-*Bgl*II fragment from the *sma-3::gfp* vector and cloned it into a *Pst*I-*Bgl*II cut *myo-2p::sma-3* minigene construct, effectively swapping the *myo-2* promoter for the *sma-3* promoter.

To test rescue of *dpy-7*, we used the same promoter fragments as above but replaced the *sma-3* minigene with genomic *dpy-7* sequence, amplified from N2 genomic DNA using the primers oGD864 (ccaaggtaccATGGAGAAGCCCAGTTCGGG) and oGD865 (ccaagagctcTTATTTCTTTCCATAACCACCACCAG), and digested with *Kpn*I and *Sac*I. The *dpy-7* promoter fragment was amplified using the primers oGD989 (aactgcagTGGCGCAAGAGGCAGTGC) and oGD990 (cggggtaccTTATCTGGAACAAAATGTAAGA).

### Generating transgenics by microinjection

*C. elegans* transgenic lines were created using standard microinjection techniques [23]. *sma-3* rescuing constructs were injected at 5–50 ng/μL, as noted in Tables and Figures, together with 30 ng/μL of either the intestinal reporter *elt-2p::tdTomato::His2B* (pJM371) or the body wall muscle reporter *myo-3p::wCherry* (kindly provided by Dr. Mei Zhen, University of Toronto) and pBlueScriptII (KS+) to a total DNA concentration of 100 ng/μL. Transgenic rescue was performed by injection directly into *sma-3* mutants, followed by screening for td-Tomato positive or wCherry-positive F1 transgenic animals, which were transferred to new plates. We specifically screened for transgenic larvae (rather than adults) to avoid biasing our selection for larger animals. We then similarly screened for F2 transgenics to establish stable lines. For rescue of *dpy-7*, transgenes were injected into wild type animals at 20 ng/μL together with 30 ng/μL of either *elt-2p::tdTomato::His2B* or *myo-3p::wCherry* and 50 ng/μL pBlueScriptII (KS+). Transgenic males were mated to *dpy-7 unc-6* mutants. Rescue of *dpy-7* was initially assayed in cross-progeny Unc males (*dpy-7 unc-6/0*). Non-Dpy non-Unc cross-progeny were also isolated, and their Unc transgenic progeny were scored for the presence or absence of the Dpy phenotype.

### Length measurements

Young adult hermaphrodites were allowed to lay eggs for 2 hours at 20°C after which they were removed and the progeny were incubated at 20°C for 96 hours. Three methods were used to obtain length measurements. In the first two methods, 96 ± 1 or 120 ± 1 hour old animals were transferred to 2% agar pads on glass slides, anaesthetized with either 5 mM levamisole or 20 mM sodium azide diluted in 1X M9 buffer and photographed at 40 × magnification under DIC optics. Levamisole was used in all experiments except those which indicate otherwise. Images were captured using a Zeiss Axio Imager.Z1 microscope with a Zeiss AxioCam MRm camera and AxioVision (4.8.1) software. Pharynx and body lengths were measured in ImageJ using segmented lines [24]. Pharynx length was measured as the distance from the posterior of the buccal cavity to the pharyngeal-intestinal valve. Calibration was achieved using a Pyser-SGI micrometer slide. Sigma Plot 12.5 was used to perform Mann–Whitney rank sum tests for statistical significance. For the final method, 96 ± 1 hour old animals were imaged directly on growth plates, in the absence of anesthetic, under a Zeiss Stemi SV11 dissecting microscope with a Canon PC1210 camera. Length measurements and statistical analysis was performed as outlined above.

### Egg to egg timing

Individual gravid hermaphrodites were picked to plates and checked periodically for egg laying. When an egg was observed to have been laid the time was recorded and the worm was picked off the plate along with any extra eggs. The laid eggs were allowed to develop at 20°C and were checked at periodic times to see if the animals had begun to lay eggs on the plate.

## Results

### The TGF-β/Sma/Mab pathway regulates pharynx length

Previous reports indicated that TGF-β/Sma/Mab signaling in the hypodermis controls body length [16,17,25]. In particular, the small body length of *sma-3* mutants was rescued to comparable levels by both hypodermal expression and the native *sma-3* promoter [16], leading to the current model that hypodermal action of the TGF-β/Sma/Mab pathway is necessary and sufficient for regulation of body length. Interestingly, pharyngeal expression of the rescuing construct using the *myo-2* pharyngeal muscle promoter also resulted in a small but statistically significant increase in body length of *sma-3* mutants [16]. Furthermore, pharynx lengths of Sma mutants at the L3 stage were found to be slightly but significantly smaller than the pharynx length of wild type N2. These two pieces of evidence suggest first, that TGF-β/Sma/Mab pathway signaling may regulate pharynx length and second, that signaling within the pharynx may contribute significantly to body length regulation.

**Figure 1:**
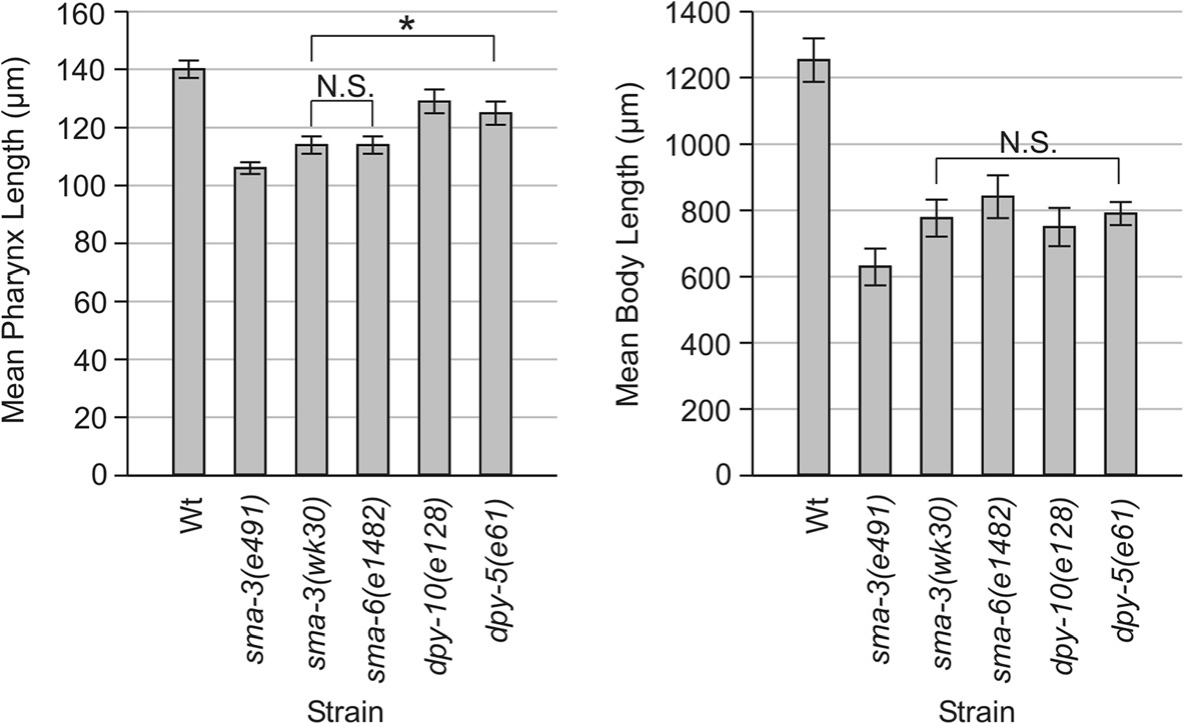
**Mean pharynx and body length measurements ± standard deviation of wild type (Wt) N2 and body size mutants at 96 ± 1 hrs AEL.** Complete data is provided in Additional file 1. * denotes a statistically significant difference of p < 0.001. All other differences in pharynx length between strains but not directly indicated on the graph are significant (p < 0.001) with the exception of N.S. (not significant). All differences in body length between strains not directly indicated on the graph are significant (p < 0.05), except where indicated by N.S.

We previously found that *sma-2(e502)* and *sma-3(e491)* mutants have adult pharynges that are 79 ± 4% and 76 ± 2% the length respectively of N2 pharynges (when measured 96 ± 1 hours after adult hermaphrodites were allowed to lay eggs for two hours at 20°C) [20]. We also find that *sma-3(wk30)* and *sma-6(e1482)* mutants have pharynges that are 81 ± 2% of N2 length (Figure 1, Additional file 1). The reduced pharynx length in these TGF-β/Sma/Mab pathway mutants suggests that pathway activity is required for pharyngeal growth, consistent with expression of pathway components in the pharynx. However, another possibility is that pharynx length is reduced as a consequence of reduced body length, specifically, that growth of the pharynx might be constrained by the smaller hypodermis. To test this latter possibility, we measured pharynx length in two hypodermal collagen mutants with reduced body length, *dpy-5(e61)* and *dpy-10(e128)* [26,27]. The body lengths of the Dpy and Sma animals are comparable. For example, *dpy-5(e61)* and *sma-3(wk30)* are 63 ± 4% and 62 ± 6% respectively of N2 body length, not a statistically significant difference (p = 0.225, Mann–Whitney Rank Sum Test on raw data). Pharyngeal lengths of *dpy-5* and *dpy-10* mutants are 89 ± 3% and 92 ± 3% of N2 length respectively, significantly greater than that of Sma mutants (p < 0.001, Mann–Whitney Rank Sum Test on raw data) (Figure 1, Additional file 1). These measurements imply that pharynx length may be partially reduced in response to smaller body length but is also positively influenced by TGF-β/Sma/Mab signaling. We therefore propose that some aspect of pharyngeal growth requires TGF-β/Sma/Mab signaling.

### The TGF-β/Sma/Mab effector SMA-3 can act in the pharynx to regulate body length

**Figure 2:**
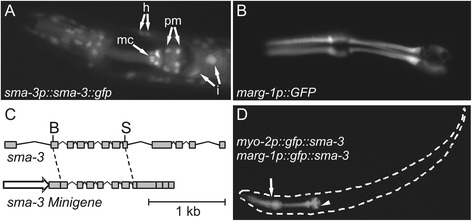
**Design and construction of pharyngeal** ***sma-3*** **minigene rescue constructs. (A)** Expression of the *sma-3p::sma-3::gfp* translational fusion. Expression is visible in the nuclei and cytoplasm of hypodermal cells (h), intestinal cells (i), pharyngeal muscles (pm) and pharyngeal marginal cells (mc). **(B)** The F47B7.7 *(marg-1)* transcriptional reporter is strongly expressed in pharyngeal marginal cells. **(C)** Top, a genomic *Bgl*II (B) - *Sal*I (S) *sma-3* fragment was cloned into a similarly digested *sma-3* cDNA clone to create the *sma-3* “minigene” (below) used for rescue experiments, under the control of various promoters (arrow); see text for details. **(D)** Pharyngeal expression of the *sma-3* minigene carrying an in-frame N-terminal GFP tag under the control of the *myo-2* and *marg-1* promoters. Expression is absent from pharyngeal gland cells (arrowhead) and weak expression is occasionally observed outside of the pharynx (arrow).

*sma-3* is reported to be expressed in the pharynx (as are other components of the pathway) [16,17], yet the individual cells in which it is expressed have not been described. We constructed a *sma-3p::sma-3::gfp* translational reporter to determine in which pharyngeal cells *sma-3* might function. As previously described [16], expression of the reporter was observed in hypodermis, intestine and pharynx. Within the pharynx, we observe expression in most or all pharyngeal muscles and marginal cells (Figure 2A). Given the expression pattern of *sma-3* in pharyngeal cells and the decreased pharynx length of *sma-3* mutants, we next asked whether pharynx length could be rescued by pharyngeal expression of *sma-3*. We performed tissue specific rescue experiments in two different *sma-3* mutant strains. The *sma-3(wk30)* mutant contains an early stop codon in *sma-3,* is predicted to be a molecular null and behaves like a genetic null allele [28]. The *sma-3(e491)* mutant contains a missense mutation in the MH2 domain of SMA-3 that is predicted to be a loss of function and genetically behaves like a strong hypomorph [28]. However, the *sma-3(e491)* allele may have dominant neomorphic properties as both mean pharynx and body length are significantly smaller in heterozygous (p < 0.001) and homozygous (p < 0.001) animals compared to the *sma-3(wk30)* nulls (Additional files 1, 2).

We attempted to rescue pharynx length by expressing a *sma-3(+)* ‘minigene’ under the control of different pharyngeal promoters (see Additional file 3 for a list of all promoters used in rescue experiments and their tissue specificity): *myo-2*, which is expressed solely in pharyngeal muscles [29] and K07C11.4, which is expressed in pharyngeal muscle, marginal cells and epithelia, the intestine, and in late stage somatic gonad [22]. We also identified *marg-1/*F47B7.7 as a marker for pharyngeal marginal cells based on our search of the Nematode Expression Pattern Database, NEXTDB [30,31]. A transcriptional reporter containing 2 kb of sequence upstream of the predicted *marg-1* start codon recapitulates this pattern of expression, showing strong expression in all marginal cells and weak, variable expression in pharyngeal epithelial cells and arcade cells and in the excretory cell of adults (Figure 2B and data not shown).

**Figure 3:**
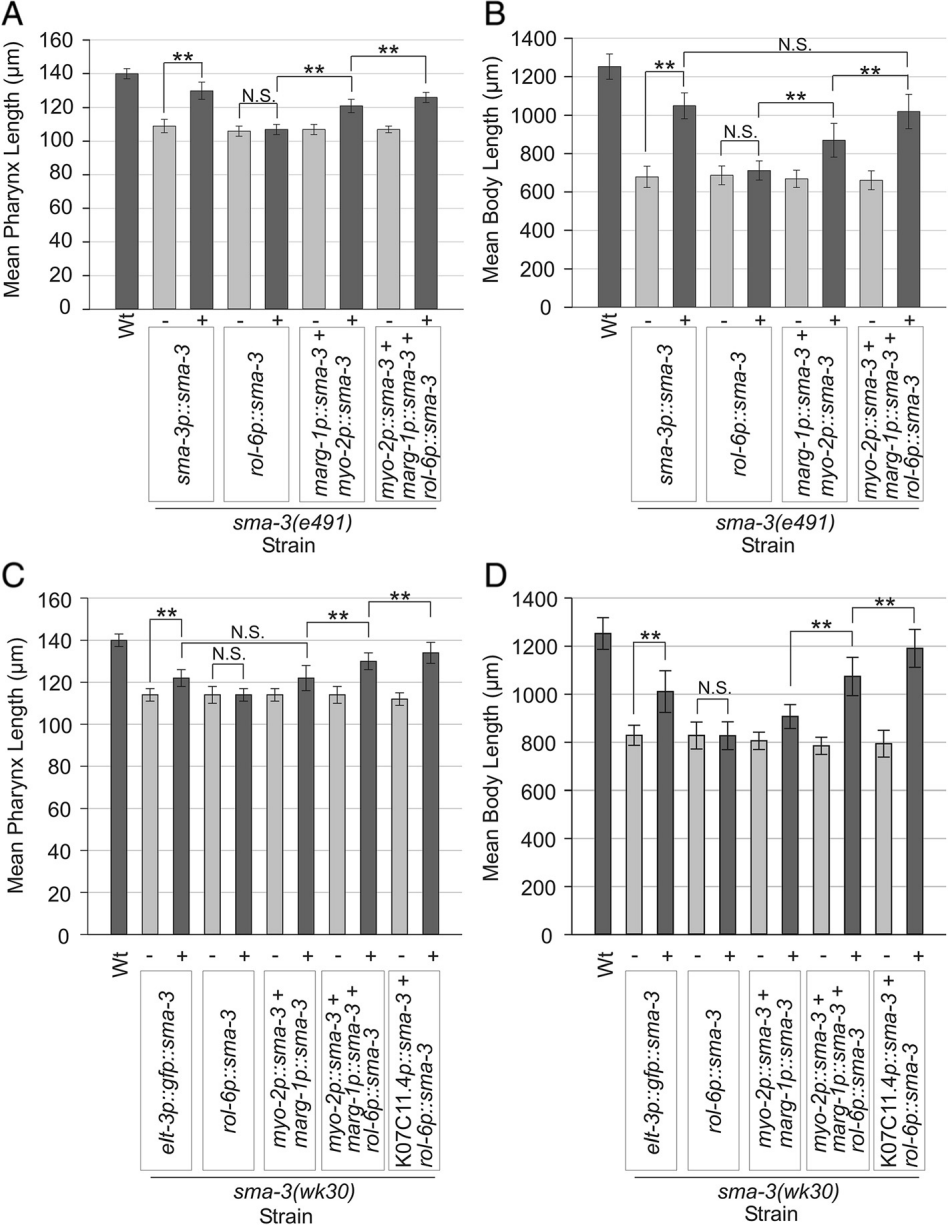
**Mean pharynx and body length measurements ± standard deviation of** ***sma-3(e491)*** **(A, B) and** ***sma-3(wk30)*** **(C, D) animals from various** ***sma-3*** **minigene rescue experiments at 96 hrs AEL.** Wild type (Wt) N2 is included for comparison. Vertical labels indicate tissue specific promoter-*sma-3* minigene fusion rescue constructs in each strain, except for *elt-3p::gfp::sma-3* which indicates the *elt-3p::gfp::sma-3* transgene (pCS223) was used. In each case, we measured animals carrying the transgenic array (+) and siblings that lacked the array (−), as indicated by presence of the transformation marker (either *elt-2p::tdTomato::His2B* or *myo-3p::wCherry*). For each transgene tested, a representative line is shown; complete data for multiple lines is provided in Additional file 5. All transgenic animal means (+) were statistically significantly different from non-transgenic sibling means (−) (p < 0.001) unless otherwise indicated. ** denotes a significant difference of p < 0.001.

The *sma-3* minigene used for rescue combines both *sma-3* cDNA and *sma-3* genomic sequence. On the one hand, the presence of introns can improve transgene expression [29,32] (Figure 2C). On the other hand, large introns can often contain control elements in *C. elegans* [21,33] and therefore these were excluded from the *sma-3* minigene to avoid affecting the tissue specific expression of the construct. The final *sma-3* minigene used in rescue experiments contains all 12 exons as well as six small introns (2–7). We first constructed *myo-2p::gfp::sma-3* and *marg-1p::gfp::sma-3* constructs, in which the minigene is fused in-frame to GFP, to verify that the *sma-3* introns did not influence the pattern of expression. As expected, in all stages we observed strong expression of these translational fusions in the pharynx with notable lack of expression in the pharyngeal glands (Figure 2D). Extremely weak expression was also observed in one to four cells just outside of the pharynx in half of the animals (n = 140, Additional file 4) but only when the exposure was increased dramatically. We noticed, however, that the GFP signal was often punctate, possibly reflecting aggregation of the fusion protein. We relied on our *sma-3* minigene (lacking GFP) because our GFP fusion had little rescuing activity (data not shown) whereas a previous report used a GFP::SMA-3 fusion to rescue *sma-3(wk30)* mutants [16]. As a positive control, we tested for rescue of *sma-3(e491)* mutants when the minigene was expressed under the control of the *sma-3* promoter (*sma-3p::sma-3*). (For all rescue experiments, the data charted in figures and referenced in the text refers to line A of each transgenic strain from the additional files; mean lengths of transgenics are presented as a percentage of non-transgenic siblings mean length). As expected, this transgene exhibited significant rescue of body length (155 ± 16% compared to 100 ± 8% for non-transgenic siblings, p < 0.001), though not to full N2 levels, possibly reflecting the artificial nature of *C. elegans* transgenic arrays and *sma-3* minigene (Figure 3, Additional file 5). We also found that pharynx length of *sma-3(e491)* mutants was significantly restored by the *sma-3p::sma-3* transgene (119 ± 6% compared to 100 ± 4% for non-transgenic siblings, p < 0.001), consistent with the expectation that pharynx length is regulated by *sma-3*. In all experiments, we measured non-transgenic siblings as a control and note that none of the strains used in these experiments displayed any strong mosaic expression of the transgenic arrays.

**Figure 4:**
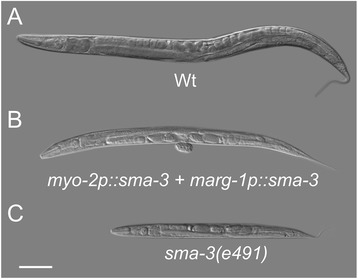
**Nomarski differential interference contrast (DIC) images of sodium azide anesthetized 96 ± 1 hrs AEL (A) Wt (N2); (B)** ***sma-3(e491)*** **mutant with pharyngeally-expressed** ***sma-3*** **minigene** ***(sma-3(e491); ivEx163[myo-2p::sma-3 marg-1p::sma-3 elt-2p::tdTomato::His2B])*** **; (C)** ***sma-3(e491)*** **mutant non-transgenic sibling of B.** Scale bar is 100 μm.

We next tested whether the *myo-2p::sma-3* and *marg-1p::sma-3* transgenes could rescue *sma-3(e491)* pharynx length to N2 levels, either alone or in combination (Figures 3A, 4, Additional file 5). *myo-2p::sma-3* rescued animals had pharynx lengths that were 110 ± 5% compared to 100 ± 3% for non-transgenic siblings and *marg-1p::sma-3* rescued animals were 107 ± 3% of N2 length compared to 100 ± 2% for non-transgenic siblings. The relative small effect of rescue on pharynx length by each of these pharyngeal transgenes was nonetheless statistically significant compared to their non-transgenic siblings (p < 0.001, Mann–Whitney Rank Sum Test on raw data). The combination of *myo-2p::sma-3* and *marg-1p::sma-3* transgenes (20 ng/μL injection mix) produced animals with an average pharynx length of 113 ± 5% compared to 100 ± 3% for non-transgenic sibling controls, a significantly greater degree of rescue compared to either alone (p < 0.001). The simple interpretation of these results is that TGF-β/Sma/Mab signaling acts within the pharynx to control pharynx length, as it also does in the hypodermis. However, we also observed an unexpected rescue of body length by pharyngeal expression of *sma-3* (Figure 3B, Additional file 5). As with rescue of pharynx length, both the *myo-2p::sma-3* and *marg-1p::sma-3* transgenes showed some rescue of body length individually, while the combination of transgenes (130 ± 16% compared with 100 ± 7% for non-transgenic siblings, 20 ng/μL injection mix) exhibited a greater rescue than either transgene alone (p < 0.01). In two variations of this rescue experiment, N2 animals, *sma-3(e491)* mutants and rescue strains were imaged in the absence of anesthetic under a dissecting microscope (Additional files 5, 6) and imaged when anesthetized by sodium azide (Figure 4, Additional file 5). These results also indicate significant but incomplete rescue of body length by pharynx specific *sma-3* minigene constructs. These findings suggest either that body length can be controlled by pharyngeal TGF-β/Sma/Mab signaling, or that our transgenic arrays are active in the hypodermis.

To rule out the possibility that the pharyngeal promoters might be active in the hypodermis, we performed two sets of experiments. First, as described above, we examined the expression of GFP-tagged versions of the transgenes, co-injected with the same transformation markers as in the rescue experiments. Strong expression was observed in the pharynx however, the exposure was also significantly increased to rule out low levels of ectopic expression. As outlined above, in overexposed images we observed variable weak GFP expression in a few cells adjacent to the pharynx (Figure 2D, Additional file 4). Quantitating the intensity in a 16bit black and white file we detect a ~25-30 fold difference between GFP reporter expression intensity in the pharynx and the faint adjacent cells, which was barely above background intensity. It is also possible that with the high exposure level that this weak signal is reflection of the pharyngeal signal from the cuticle. We note that our injection mixes did not contain any other hypodermally expressed genes that might have influenced expression of the *sma-3* transgenes such as the hypodermal *rol-6* (pRF4) transformation marker [23,34,35]. Instead, we used either an intestinal reporter or body wall muscle reporter to identify transgenic animals. A second experiment tested whether the combination of *myo-2* and *marg-1* promoters might be active in the hypodermis by testing rescue of the hypodermal mutant *dpy-7* to further rule out ectopic expression from the pharyngeal promoters*.*

The *dpy-7* gene encodes a collagen that is expressed in the hypodermis and by its nature is expected to act autonomously in the hypodermis to affect body length (and shape) [36,37]. We tested whether the combination of the pharyngeal *myo-2* and *marg-1* promoters might be unexpectedly active in hypodermis by seeing if they could drive expression of *dpy-7(+)* to rescue the *dpy-7(e88)* mutant phenotype. The combination of *myo-2p::dpy-7* and *marg-1p::dpy-7* transgenes did not exhibit any rescue of the mutant phenotype (Table 1, Additional file 7). Most worms with the *rol-6p::dpy-7* construct appeared rescued however 9% displayed an intermediate phenotype between Dpy and wild type suggesting that partial rescue occurred in this case. In contrast, the *dpy-7p::dpy-7* transgene rescued 100% of mutant animals assayed. We therefore conclude from these two lines of evidence that the combination of the *myo-2* and *marg-1* promoters does not significantly activate expression of transgenes in the hypodermis. We propose instead that pharyngeal expression of *sma-3* can partially rescue the small body length of *sma-3* mutants.

**Table 1:** **Frequency of Dpy phenotype rescue**

**Transgene**	**% non-dpy**	**% intermediate**	**n**
*dpy-7p::dpy-7*	100%	0%	33
*myo-2p::dpy-7* + *marg-1p::dpy-7*	0%	0%	21
*rol-6p::dpy-7*	74%	9%	46

Rescue of *dpy-*7 mutants by different *dpy-7*–expressing transgenes, expressed as the percentage of transgenic animals showing rescue of the dpy phenotype (‘% non-dpy’). Representative animals are shown in Additional file 7.

### TGF-β/Sma/Mab signaling can act in multiple tissues to control overall body length

To further test the tissue specificity of *sma-3* in body length regulation, we performed rescue tests of *sma-3(e491)* mutants using *rol-6p::sma-3* and *dpy-7p::sma-3* transgenes. Importantly, these constructs use the same promoter sequences used to rescue *dpy-7* as a control above, confirming that the promoters are functional in the hypodermis. Surprisingly, we found that neither transgene had rescuing activity in *sma-3(e491)* mutants when injected alone (Figure 3A, B, Additional file 5, data not shown). One possibility for the weaker rescue is that the relative dose of the transgene might be too low. We therefore increased the concentration of *rol-6p::sma-3* used in our injection mixes. However, increasing the *rol-6p::sma-3* transgene dose did not enhance rescue of *sma-3(e491)* mutants (Additional file 5). We do note that others have also reported lack of rescuing activity with the *rol-6* promoter for LON-2 (but not with another hypodermal promoter), a protein proposed to bind to the DBL-1 ligand to function in the hypodermis as a negative regulator of the TGF-β/Sma/Mab pathway [38].

Another possibility is that our *rol-6p::sma-3* transgene is not functional or has reduced function. However, we find that our hypodermal *sma-3* transgene is functional when used in combination with the pharynx-expressed *sma-3* transgenes. We created transgenic lines carrying *myo-2p::sma-3, marg-1p::sma-3* and *rol-6p::sma-3* in the *sma-3(e491)* background and compared their pharyngeal and body length rescuing ability to the combination of the pharyngeal promoters alone. We found that this combination of pharyngeal and hypodermal transgenes leads to an average pharynx length of 118 ± 4% compared to 100 ± 2% for non-transgenic siblings, and an average body length of 154 ± 18% compared to 100 ± 7% for non-transgenic siblings (Figure 3A, B, Additional file 5). This pharyngeal and hypodermal combination of transgenes resulted in significantly greater rescue of both pharynx and body length in *sma-3(e491)* mutants than seen with the pharyngeal transgenes alone (p < 0.001) (Figure 3A, B, Additional file 5). Furthermore, the combination of pharyngeal (*myo-2p::sma-3* and *marg-1p::sma-3)* and hypodermal (*rol-6p::sma-3)* transgenes was able to rescue body length of *sma-3(e491)* mutants to the same extent as the native *sma-3p::sma-3* transgene (154 ± 18% and 155 ± 16% respectively of their non-transgenic siblings, p = 0.173), though the difference in pharynx lengths was still significant (p < 0.001).

As an additional test of the generality of these results, we performed similar tissue specific rescue experiments with the *sma-3(wk30)* strain as all of the above tests were done with the *e491* allele. We independently assayed hypodermal rescuing activity of three constructs: our own *rol-6p::sma-3* minigene; as well as two constructs (kindly provided by Dr. Cathy Savage-Dunn) that have been reported to rescue *sma-3(wk30)* mutants, *dpy-7p::gfp::sma-3* (pCS226*)* and *elt-3p::gfp::sma-3* (pCS223) [16]. Similar to the results observed for the *sma-3(e491)* background, we did not see significant rescue of the pharynx or body length phenotypes of *sma-3(wk30)* with the *rol-6p::sma-3* transgene (Figure 3C, D, Additional file 5). However, hypodermal expression using the *elt-3p::gfp::sma-3* transgene did significantly rescue pharynx length (107 ± 5% compared to 100 ± 3% for non-transgenic siblings, p < 0.001) and body length (122 ± 12% compared to 100 ± 5% for non-transgenic siblings, p < 0.001). Again, we found that the combination of the pharyngeal (*myo-2p::sma-3, marg-1p::sma-3*) and hypodermal (*rol-6p::sma-3*) transgenes provided significantly greater rescue of *sma-3(wk30)* mutants by 96 ± 1 hours after egg laying compared to the pharyngeal transgenes alone (114 ± 5% of non-transgenic sibling pharynx length compared to 107 ± 6% for pharyngeal transgenes alone, p < 0.001 and 137 ± 12% of non-transgenic sibling body length compared to 113 ± 8% for pharyngeal transgenes alone, p < 0.001) (Figure 3C, D, Additional file 5). Thus, our results do not appear to depend strongly on allele-specific effects.

We also tested whether a different pharyngeal promoter could replace the combination of *myo-2* and *marg-1* in rescue of *sma-3(wk30)* mutants. We used the well-characterized pharyngeal promoter from K07C11.4, which is active in pharyngeal muscle, marginal cells and epithelial cells, as well as in the intestine and late-stage somatic gonad, but is not expressed in the hypodermis [22]. On its own, K07C11.4*p::sma-3* had little rescuing activity (data not shown). However, the combination of K07C11.4*p::sma-3* and *rol-6p::sma-3* showed significant rescue of both pharynx length (120 ± 5% compared to 100 ± 3% for non-transgenic siblings, p < 0.001) and body length (150 ± 15% of N2 length compared to 100 ± 7% for non-transgenic siblings, p < 0.001) (Figure 3C, D, Additional file 5). This K07C11.4*p::sma-3* and *rol-6p::sma-3* combination had significantly greater pharynx and body length rescue than that observed with the combination of *myo-2p::sma-3, marg-1p::sma-3,* and *rol-6p::sma-3* (p < 0.001)*.* Thus, we conclude the rescue of body length by pharyngeal *sma-3* minigene constructs is not a unique feature of *myo-2* and *marg-1* pharyngeal promoters.

We draw three conclusions from these results. First, the hypodermal promoter *rol-6* is capable of driving *sma-3* expression and this expression contributes to rescue of *sma-3* mutants, at least when present in an extrachromosomal array in combination with other rescue constructs. Second, hypodermal expression of *sma-3* can influence pharyngeal length, just as pharyngeal expression can influence body length. Thirdly, *sma-3* can function in both of these tissues to promote normal growth.

During the course of these experiments it was noticed that some *sma-3* mutant animals were developmentally delayed compared to wild type animals by the time of imaging (96 ± 1 hours after egg laying). We were concerned that this could affect how we interpret the results so we characterized the mean egg-to-egg time for three strains: wild type N2, the *sma-3(e491)* mutant and the pharyngeal *sma-3(e491)* rescue strain *myo-2p::sma-3 + marg-1p::sma-3* (30 ng/μL). As seen in Table 2, 100% of wild type animals and 91% of transgenic pharyngeal rescue animals had begun egg laying by 96 hours. In contrast, only 68% of *sma-3(e491)* animals had begun laying eggs by 96 hours. This suggests that we are over estimating the quantity of rescue with each of our transgenic strains. However, we do note that 35% of wild type worms had begun laying eggs by 72 hrs compared to 0% of the transgenic pharyngeal rescue worms. This result indicates that while these pharyngeal rescue transgenic animals develop somewhat ahead of their non-transgenic mutant siblings, they were also developmentally delayed relative to wild type. This result is consistent with the partial in-between Sma phenotype rescue observed with this strain. To account for this feature of the *sma-3* mutant phenotype, as all worms had begun egg laying by 114 hours we repeated our length analysis at 120 hours.

**Table 2:** **Percentage of Wt and** ***sma-3(e491)*** **animals (+ or - pharyngeal** ***sma-3*** **minigene constructs) that had laid an egg by the time point indicated (hrs after initial egg laid)**

**Strain/time point**	**48 hrs**	**66 hrs**	**72 hrs**	**90 hrs**	**96 hrs**	**114 hrs**	**120 hrs**	**n**
Wt	0%	8.7%	34.8%	100%	100%	100%	100%	23
*sma-3(e491)*	0%	0%	0%	20%	68%	100%	100%	25
*sma-3(e491)*	0%	0%	0%	42.9%	66.7%	100%	100%	21
*myo-2p::sma-3 + marg-1p::sma-3 (−)*								
*sma-3(e491)*	0%	0%	0%	88.2%	91.1%	100%	100%	34
*myo-2p::sma-3 + marg-1p::sma-3 (+)*								

**Figure 5:**
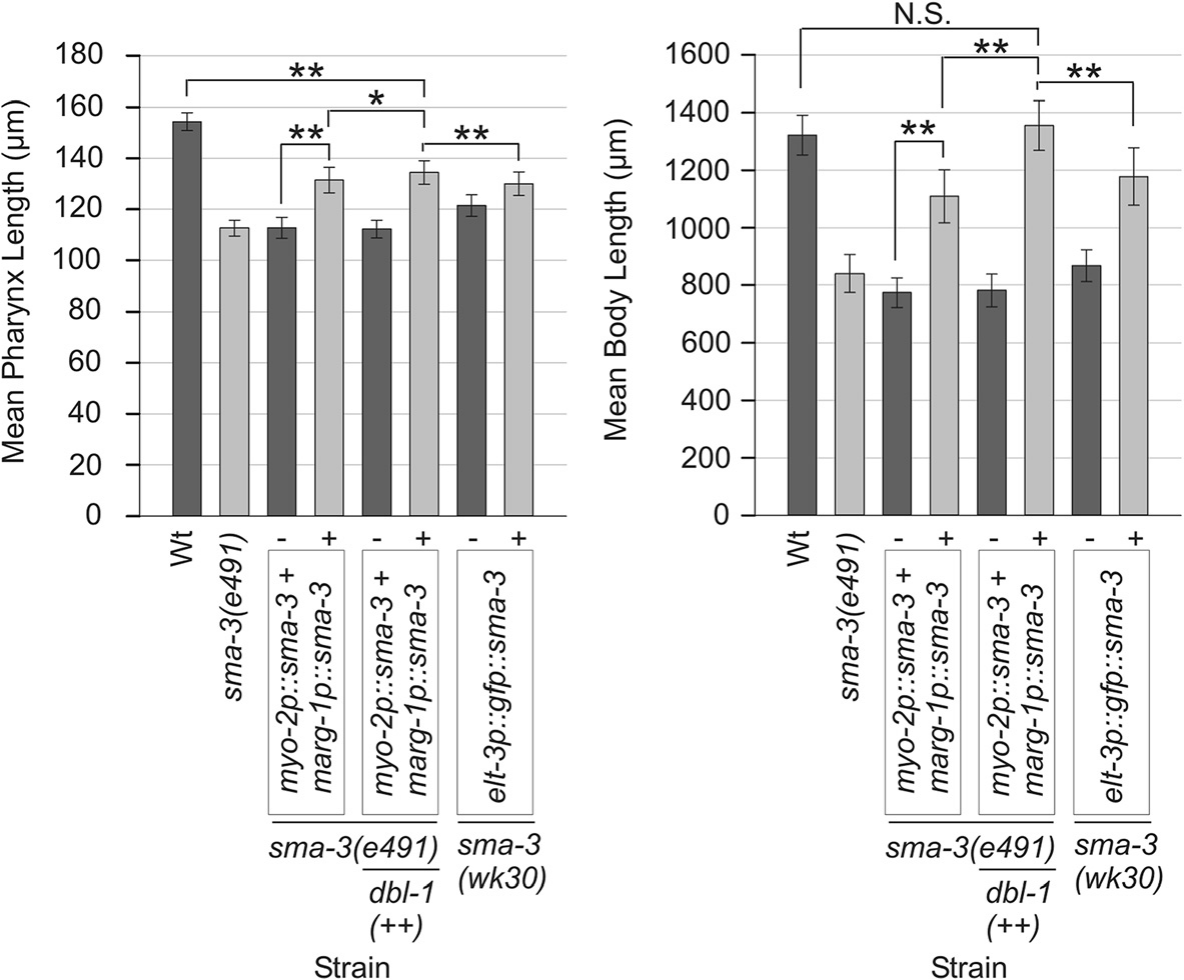
**Mean pharynx and body length measurements ± standard deviation of sodium azide anesthetized wild type (Wt) and** ***sma-3*** **mutant animals at 120 ± 1 hrs AEL from various** ***sma-3*** **minigene rescue experiments.** Vertical labels indicate tissue specific promoter-*sma-3* minigene fusion rescue constructs in each strain, except for *elt-3p::gfp::sma-3* which indicates the *elt-3p::gfp::sma-3* transgene (pCS223) was used. In each case, we measured animals carrying the transgenic extrachromosomal array (+) and siblings that lacked the array (−), as indicated by a transformation marker (either *elt-2p::tdTomato::His2B* or *myo-3p::wCherry*). Horizontal labels indicate genetic background. All transgenic animal means (+) were statistically significantly different from non-transgenic sibling means (−) (p < 0.001). ** denotes significant differences of p < 0.001, * denotes significant differences of p < 0.05.

We observed partial rescue of both pharynx length and body length with the pharyngeal promoters driving the *sma-3* minigene (*sma-3(e491)* background) as well as the *elt-3::gfp::sma-3* transgene (*sma-3(wk30)* background) (Figure 5, Additional file 5) which is consistent with our previous results. Furthermore, a statistical significant difference is observed between *sma-3(e491)* mutants at 120 ± 1 hours and pharyngeal rescued transgenic animals at 96 ± 1 hours for both pharynx length (110 ± 5% compared to 100 ± 3%, p < 0.001) and body length (122 ± 11% compared to 100 ± 8%, p < 0.001) (Additional file 5). These results confirm that pharyngeal signaling is partially rescuing pharynx length and body length in *sma-3(e491)* mutants.

As an extension of these results, we crossed in the *ctIs40[dbl-1(+) surf-5::gfp]* integrated multicopy array [14] to our pharyngeal rescue strain to create JM228: ctIs40[*dbl-1(+) sur-5::gfp*] *X; sma-3(e491) III; ivEx163[myo-2p::sma-3 marg-1p::sma-3 phat-1p::yfp elt-2p::tdTomato::His2B]*. In a wild type background the *ctIs40* insertion results in a Lon phenotype due to the overexpression of the TGF-β/Sma/Mab pathway ligand *dbl-1* [14]. However, in the newly constructed strain the downstream *sma-3(e491)* mutation should be epistatic to *dbl-1* overexpression. As expected, all worms with the *dbl-1(+)* array that did not get the extra chromosomal pharyngeal *sma-3* rescue array (as determined by the lack of extra chromosomal array reporter expression) had small mean pharynx length and body length (Figure 5, Additional file 5). Surprisingly, the animals that carried the extra chromosomal pharyngeal *sma-3* rescue array and *dbl-1(+)* array insertion had fully rescued body length compared to wild type (p = 0.132) but not pharynx length (p < 0.001) (Figure 5, Additional file 5). The increase in rescue that is observed with the elevated dose of *dbl-1* ligand validates our conclusion it is TGF-β/Sma/Mab pathway signaling in the pharynx that is rescuing body length in these animals and not an independent function of SMA-3. We conclude from this experiment that pharynx length is regulated by TGF-β/Sma/Mab pathway signaling from both within and outside the pharynx.

Expression of *sma-3* in multiple tissues might rescue *sma-3(e491)* and *sma-3(wk30)* mutants for two reasons. Each tissue could make a distinct contribution to growth, for example, by expressing distinct secreted signals that act on different downstream components. Alternatively, the effect on rescue could be quantitative rather than qualitative whereby simply increasing the dose of *sma-3* in a single tissue may be sufficient to increase rescue. As noted above, increasing the relative concentration of hypodermal *sma-3* transgenes had no observable effect on rescue of *sma-3(e491)* mutants. Likewise, reducing the dose of *sma-3* in each of three tissues (pharyngeal muscle, pharyngeal marginal cells and hypodermis) only slightly reduced rescuing activity (Additional files 1, 2, 5, 8). While we cannot interpret whether this effect reflects a qualitative difference (e.g. tissue-specific targets) or quantitative difference (amount of downstream signal produced by the tissues) or a combination of both, the results support the conclusion that *sma-3* can act in multiple tissues to control growth.

## Discussion

### The TGF-β/Sma/Mab pathway regulates pharynx length

In a previous study on the morphology of the pharynx, we identified a number of Sma mutants with decreased pharynx lengths compared to wild type, including members of the TGF-β/Sma/Mab signaling pathway *sma-*2 and *sma-3* [20]. Here we report that mutants of TGF-β/Sma/Mab pathway components have significantly smaller pharynx lengths compared to Dpy mutants of similar body length. The pharynges of Dpy mutants are also significantly smaller than wild type suggesting that growth of this organ is limited by the length of the entire animal. Thus, pharynx length appears to be determined partly by positive TGF-β/Sma/Mab signaling but is also dependent on the overall body length of the animal. It was interesting that signaling in the pharynx never resulted in full rescue of pharynx length even when body length was completely rescued. This result implies that TGF-β/Sma/Mab signaling from outside the pharynx must also play a role in determining pharynx length.

It is important to note the similarity in growth control of the pharynx and the hypodermis, where both organs utilize positive TGF-β/Sma/Mab signaling to regulate organ and overall body length. Furthermore, pharynx and body length can be restricted by morphology defects in components of the surrounding extracellular cuticle. Previous study of mutations in extracellular matrix (ECM) components (including the cuticle collagen *dpy-7*) and membrane proteins revealed a role in pharynx morphology and a twisted pharynx (Twp) phenotype [39,40]. The authors observed bending of contractile arrays in pharyngeal muscles and hypothesized that during normal pharyngeal growth, the defective ECM must restrict these arrays. It is unclear if Twp animals have reduced pharynx lengths but if so it would be a noteworthy parallel between pharynx growth and overall body growth. It would be interesting to see if there are any common ECM or membrane components as downstream targets of pharyngeal and hypodermal TGF-β/Sma/Mab pathway signaling.

The hypodermis undergoes post-embryonic endoreduplication and it is has been suggested that a primary function of TGF-β/Sma/Mab pathway signaling is to positively regulate this event to achieve body size regulation [41]. On the other hand, analysis of potential downstream targets of the pathway did not reveal a large number of cell cycle regulators [10] and it is still known which direct downstream targets of the TGF-β/Sma/Mab pathway are most critical for regulating body size. In contrast, the pharynx does not undergo any cell number or ploidy changes during post embryonic development [4]. It would be interesting to see if this pathway mediates cell and tissue growth via different downstream targets in the pharynx vs the hypodermis.

### Activity of the TGF-β/Sma/Mab pathway in multiple tissues can control body length

The results described here support a model in which downstream effectors of TGF-β/Sma/Mab signaling can act in both the pharynx and hypodermis to influence overall body length in *C. elegans*. Multiple lines of evidence support this model. First, expression of *sma-3* under the control of different pharyngeal promoters (either *myo-2* or *marg-1* alone or in combination) is able to partially rescue body length of *sma-3* mutants. These promoters are not active in the hypodermis, based on the lack of expression of GFP reporters and inability to rescue the hypodermal collagen mutant *dpy-7(e88)*. Second, we find that simultaneous expression of *sma-3* in both the hypodermis and pharynx provides stronger rescue of body length than when *sma-3* is expressed in either tissue alone suggesting that both can contribute to normal growth in an additive manner. Third, overexpression of the *dbl-1* ligand with only pharyngeal signaling results in complete rescue of body length indicating that while likely insufficient in wild type situations, pharyngeal signaling is capable of regulating body length. Finally, components of the TGF-β/Sma/Mab pathway, including SMA-3, are expressed in the pharynx and hypodermis, consistent with the proposed function of this pathway in these tissues. Interestingly, at least one other TGF-β/Sma/Mab component has been demonstrated to act in the pharynx. Body length (and width) of *daf-4* mutants, the Type II TGF-β receptor, can be rescued by expression of *daf-4* in pharyngeal muscle under the control of the *myo-2* promoter [42], consistent with our findings for *sma-3*.

Our results differ from previous reports that hypodermal expression of TGF-β/Sma/Mab components is sufficient for body length rescue [16,17,25]. This difference may only represent variation in the degree of rescue, as we do observe partial rescue with the hypodermal *elt-3p::gfp::sma-3* transgene, though not to the extent previously reported. It is possible that these disparities may also reflect differences in generation of transgenic lines. Likewise, hypodermal expression of other components (*sma-6, sma-10* and *drag-1*) is sufficient for complete rescue [17,25,43]; though this does not preclude contributions from other tissues. We did not observe any rescue with the *rol-6p::sma-3* and *dpy-7p::sma-3* transgenes when injected alone however, cuticle collagen gene expression (including *rol-6*) is known to cycle in relation to molts [44] and as such the *rol-6p* and *dpy-7p* hypodermal promoters may not drive sufficient expression of the rescuing transgene at necessary times in development to achieve rescue. Additionally, the *dpy-7p::sma-3* transgene appeared to have integrated into the genome. The lack of rescue observed in this strain may be explained by an integration event that disrupted an important gene as this strain appeared sick relative to the other transgenic strains generated. Previous work demonstrated that a *sma-3* transgene under control of the *myo-2* promoter weakly but significantly rescued growth of *sma-3(wk30)* mutants, similar to what we observe [16]. However, no tests were performed with the combination of *myo-2* and other pharyngeal promoters, i.e. simultaneous expression in both marginal cells and muscles was not tested as we did here, which resulted in an obvious partial rescue of body length.

The rescuing activity of pharyngeal and hypodermal promoters when used in combination was consistently more robust than either promoter alone. In the *sma-3(e491)* background, this combination of transgenes rescued body length to the same extent as the native *sma-3* promoter construct suggesting that both tissues can contribute to body length regulation. Interestingly, the K07C11.4 promoter, which drives expression in the pharynx and late stage somatic gonad did not rescue by itself but in combination with the *rol-6p::sma-3* transgene almost completely rescued the *sma-3(wk30)* body length phenotype to N2 levels. We do note that complete rescue of the *sma-3* body length phenotype to N2 length was only observed when the *dbl-1* ligand was over expressed. It is unclear what underlies the differences in rescue activity of the various extrachromosomal arrays tested here. One source of variation could be the copy number of each transgene present in each extrachromosomal array and the level of expression of the *sma-3* minigene from each array. Furthermore, the neomorphic properties of the *sma-3(e491)* allele may interfere with the rescuing activity of the *sma-3* minigene. This could account for the observation of almost complete rescue of body length to N2 levels with the K07C11.4*p::sma-3* and *rol-6p::sma-3* transgene combination in the *sma-3(wk30)* background but the lack of complete rescue with the *sma-3p::sma-3* transgene in the *sma-3(e491)* background.

## Conclusions

Given the developmental delay observed in animals homozygous for *sma-3* mutant alleles and the artificial nature of *C. elegans* extrachromosomal arrays it is difficult to quantitate exactly how much contribution TGF-β/Sma/Mab signaling in the pharynx makes to regulation of body length. Certainly the only case where full rescue of body length was achieved was with high levels (presumed to be greatly in excess of wild type levels) of the *dbl-1* ligand present. Taken together, using our results presented here and those of previous studies on tissue specific regulation of body length [16,17,42], we make the following conclusions about pharynx length and body length regulation by TGF-β/Sma/Mab signaling. TGF-β/Sma/Mab signaling in the pharynx is capable of contributing to pharynx length and body length regulation but this signaling is not sufficient or necessary to facilitate wild type pharynx length or body length.

**Figure 6:**
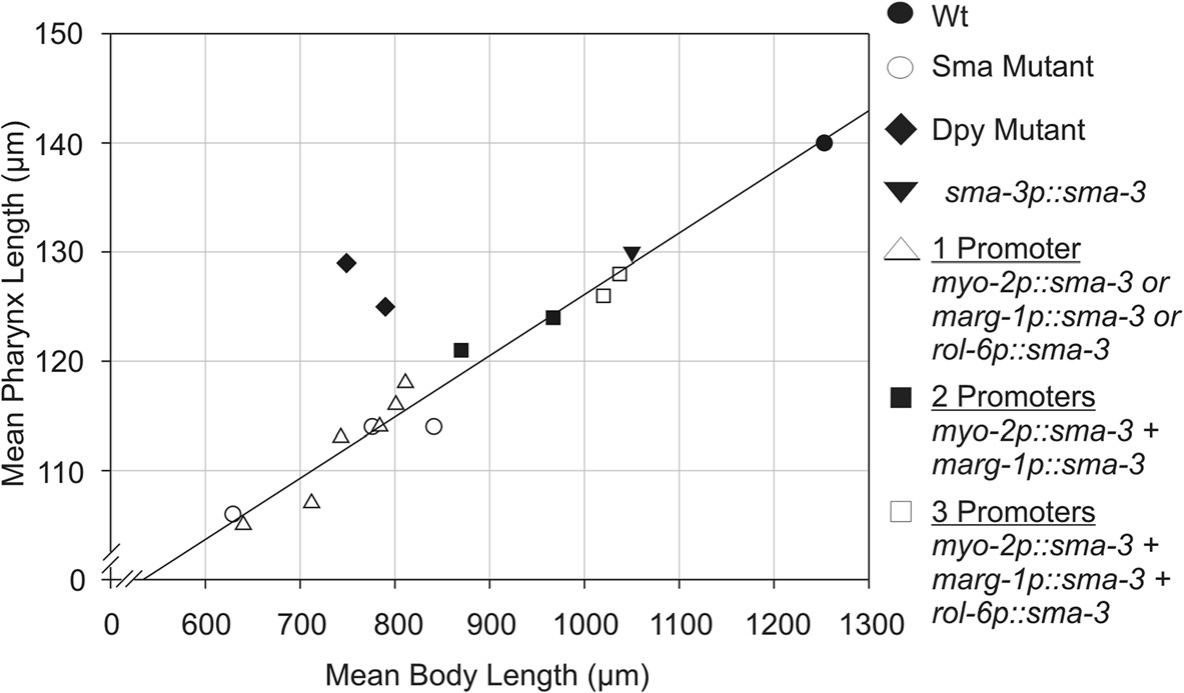
**Linear relationship between pharynx length and body length in mutants of the TGF-β/Sma/Mab pathway and strains carrying various** ***sma-3*** **rescuing extrachromosomal arrays in the** ***sma-3(e491)*** **mutant background.** One promoter refers to cases where only a single *sma-3* minigene rescue construct was used (either *myo-2p::sma-3*, *marg-1p::sma-3*, or *rol-6p::sma-3*). Two and three promoters refer to strains carrying all indicated transgenes. Wild type (Wt) N2 is included for comparison. The correlation coefficient for the linear model is R^2^ = 0.97. Notably, *dpy-5* and *dpy-10* mutants (black diamonds; not included in the linear model) do not fall on the line.

Coordination of growth is an interesting feature of all of the animals examined here. We note a strong linear correlation between pharynx and body length, except in Dpy mutants, in which signaling is presumably normal but body length is reduced due to defects in hypodermal collagen (Figure 6). Consistent with signaling between tissues, animals in which manipulation of TGF-β/Sma/Mab signaling results in uncoupled hypodermal and pharynx lengths were not observed (i.e. we have not seen small animals with big pharynges or vice versa). Furthermore, organs of Dpy mutants such as the pharynx and gonad often appear compressed compared to those of TGF-β/Sma/Mab pathway mutants which appear more proportional to the overall body length of the animal [11]. This suggests that TGF-β/Sma/Mab signaling in the pharynx and hypodermis may be coordinating growth of many tissues within the animal.

## Additional files

Additional file 1: Table S1. Pharynx and Body Lengths of Various Strains. Measurements of pharynx and body length of wild type (Wt) N2 and various mutant strains, expressed as mean (± standard deviation). These same data are charted in Figure 1 and Additional file 2: Figure S1. ^a^data previously reported in (Raharjo et al., [20]). Additional file 2: Figure S1. Graphs of Pharynx and Body Lengths of Wt and Various sma-3 Mutant Strains. Mean pharynx and body length measurements ± standard deviation of Wild type (Wt) N2, sma-3 mutants and sma-3 heterozygotes. Complete data is provided in Additional file 1. * denotes statistically significant differences of p<0.001. All other differences in pharynx and body lengths between strains not directly indicated on the graphs are significant (p<0.001). Additional file 3: Table S2. Tissue Specificity of Promoters Used to Drive Rescue Constructs. List of promoters used to drive sma-3 minigene rescue constructs and the tissues they are active in. Additional file 4: Figure S2. Pharyngeal Expression of the sma-3 Minigene with in-frame GFP tag. Pharyngeal expression of the sma-3 minigene carrying an in-frame N-terminal GFP tag under the control of the myo-2 and marg-1 promoters. Very weak expression is occasionally observed outside of the pharynx in some animals (arrowheads). Additional file 5: Table S3. Pharynx and Body Lengths of Strains Imaged Using Various Anesthetics at Different Time Points After Egg Laying During Development. Measurements of pharynx and body length of sma-3(e491) and sma-3(wk30) animals carrying different sma-3 minigene constructs (mean ± standard deviation). ‘DNA Conc’ is the concentration of the transgene used in the original microinjections. ‘Line’ indicates independent transgenic lines (arbitrarily named), from which we measured both non-transgenic (−) and transgenic (+) animals. Some of these data are charted in Figures 3 and 5, Additional files 6 and 7. Additional file 6: Figure S3. Graphs of Pharynx and Body Lengths of Various Strains in the Absence of Anesthetic. Mean body length measurements ± standard deviation (measured under a dissecting microscope in the absence of anesthetic) of Wild type (Wt) N2, sma-3(e491) and sma-3(e491) animals from various sma-3 minigene rescue experiments. Vertical labels indicate tissue specific promoter-sma-3 minigene fusion rescue constructs in each strain. In each case, we measured animals carrying the transgenic array (+) and siblings that lacked the array (−), as before. Complete data for all lines is provided in Additional file 5. All transgenic animal means (+) were statistically significantly different from non-transgenic sibling means (−) (p<0.001). * denotes significant differences of p<0.001. All other differences in body lengths between strains not directly indicated on the graphs are significant (p<0.05). Additional file 7: Figure S4. Images of dpy-7 Rescue Experiment Phenotypes. Rescue of dpy-7 mutants by different transgenes. (A) Rescue of dpy-7 by a dpy-7p::dpy-7 transgene. (B) Expression of dpy-7 under the control of the pharyngeal promoters myo-2 and marg-1 does not rescue the dpy-7 phenotype. (C) Rescue of dpy-7 by a rol-6p::dpy-7 transgene. Complete data is provided in Table 1. Scale bar is 150 μm. Additional file 8: Figure S5. Reduction of transgene dose has only minor effects on sma-3 rescue. Measurements are means ± standard deviation. Transgenic lines were established using injection mixes containing myo-2p::sma-3, marg-1p::sma-3 and rol-6p::sma-3, each at a concentration of either 20 ng/μL or 5 ng/μL, as indicated. A and B indicate independently generated transgenic lines. All transgenic animal means (+) were statistically significantly different from non-transgenic sibling means (−) (p<0.001). All differences in pharynx length not directly indicated on the graph are significant (p<0.001), except where indicated by N.S. * denotes significant differences of p<0.05. All other differences in body length not directly indicated on the graph are significant (p<0.05) except where indicated by N.S. Complete data for multiple lines is provided in Additional file 5.
