# Supplementary figures and images for: TGF-β signaling can act from multiple tissues to regulate C. elegans body size
Source: BMC Dev Biol. 2014 Dec 6;14:43. doi: 10.1186/s12861-014-0043-8 (PMC4278669; doi:10.1186/s12861-014-0043-8)

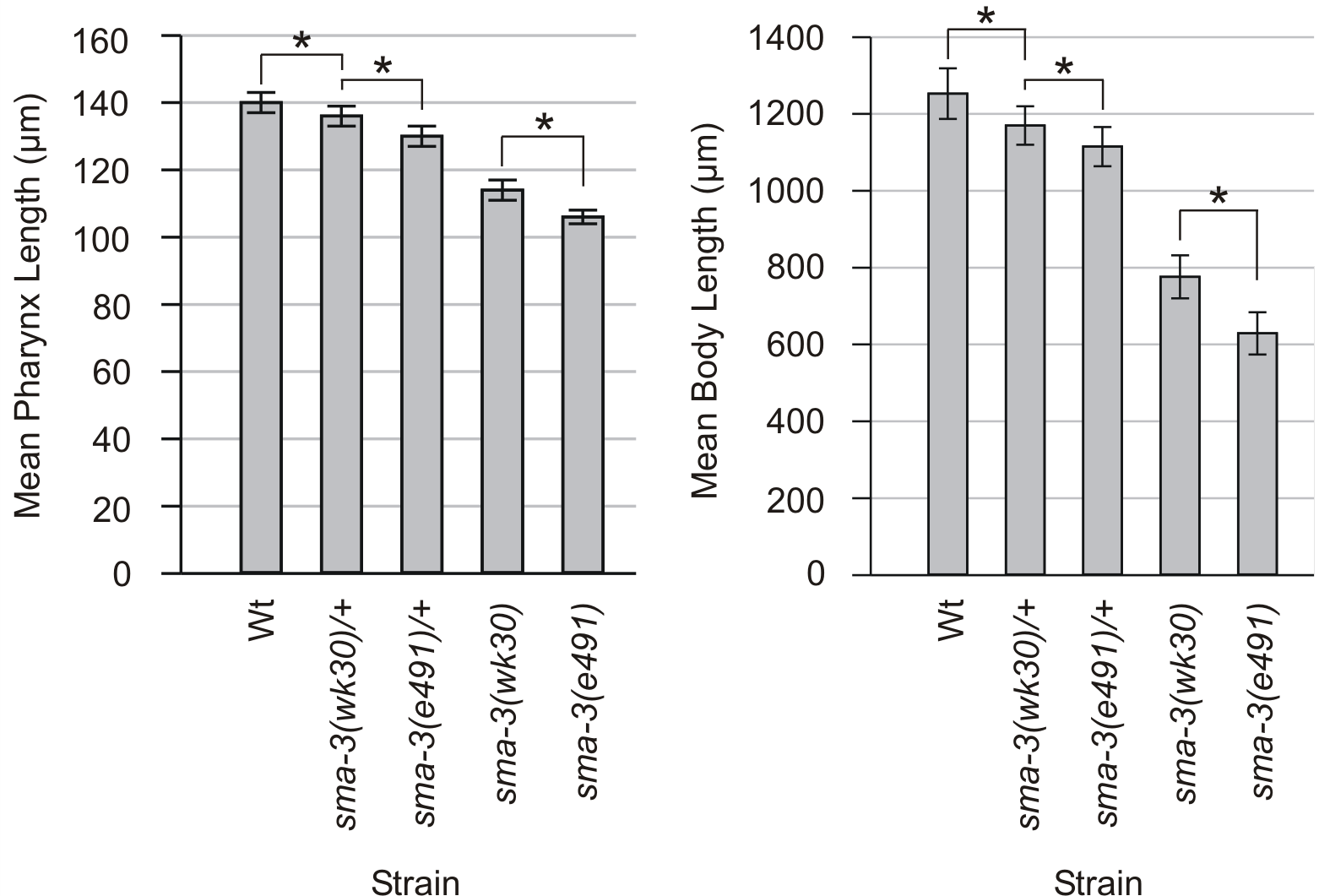

Supplement: Additional file 2: Figure S1. — Graphs of Pharynx and Body Lengths of Wt and Various sma-3 Mutant Strains. Mean pharynx and body length measurements ± standard deviation of Wild type (Wt) N2, sma-3 mutants and sma-3 heterozygotes. Complete data is provided in Additional file 1. * denotes statistically significant differences of p<0.001. All other differences in pharynx and body lengths between strains not directly indicated on the graphs are significant (p<0.001). [file 12861_2014_43_MOESM2_ESM.tiff]

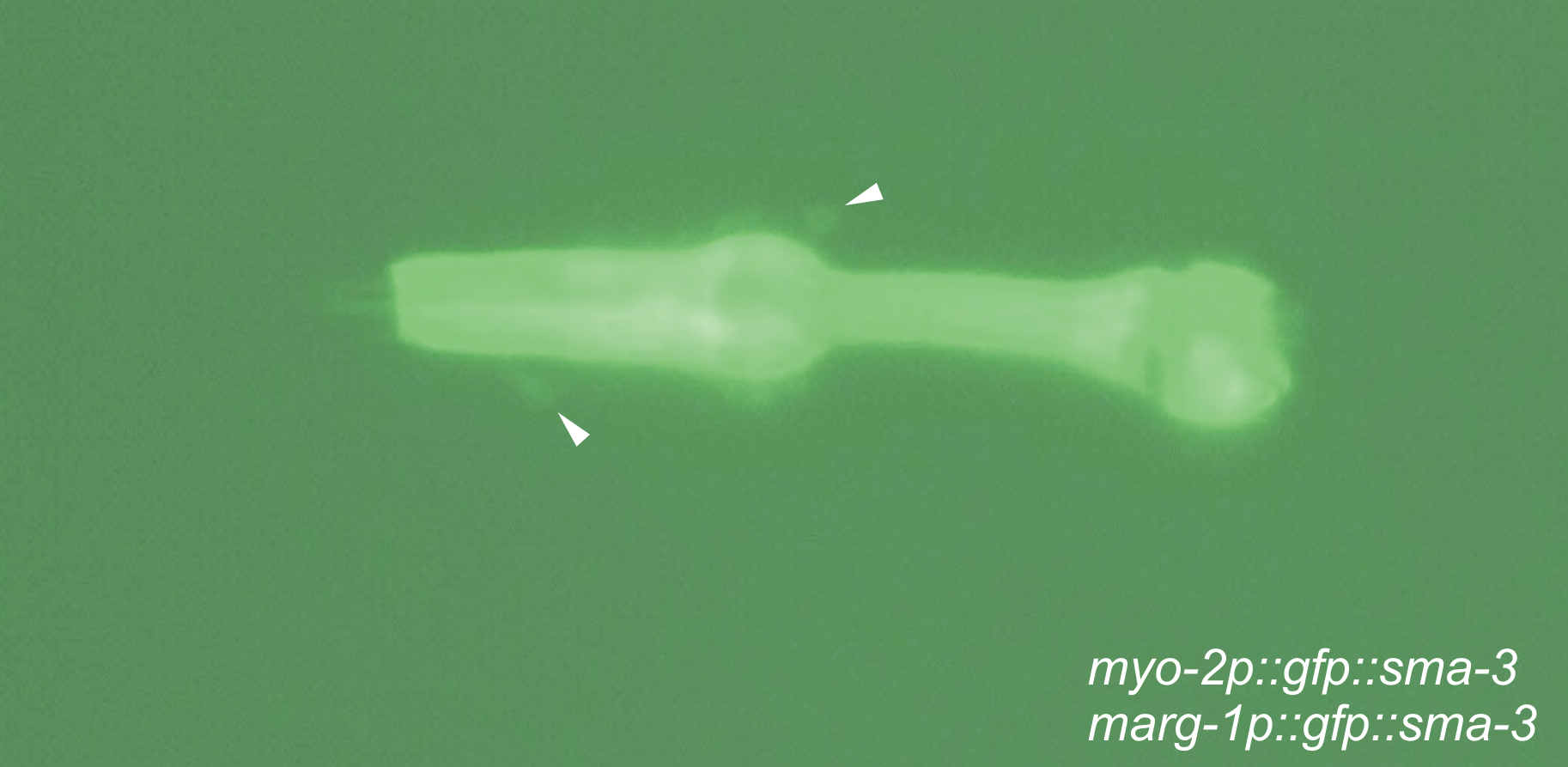

Supplement: Additional file 4: Figure S2. — Pharyngeal Expression of the sma-3 Minigene with in-frame GFP tag. Pharyngeal expression of the sma-3 minigene carrying an in-frame N-terminal GFP tag under the control of the myo-2 and marg-1 promoters. Very weak expression is occasionally observed outside of the pharynx in some animals (arrowheads). [file 12861_2014_43_MOESM4_ESM.tiff]

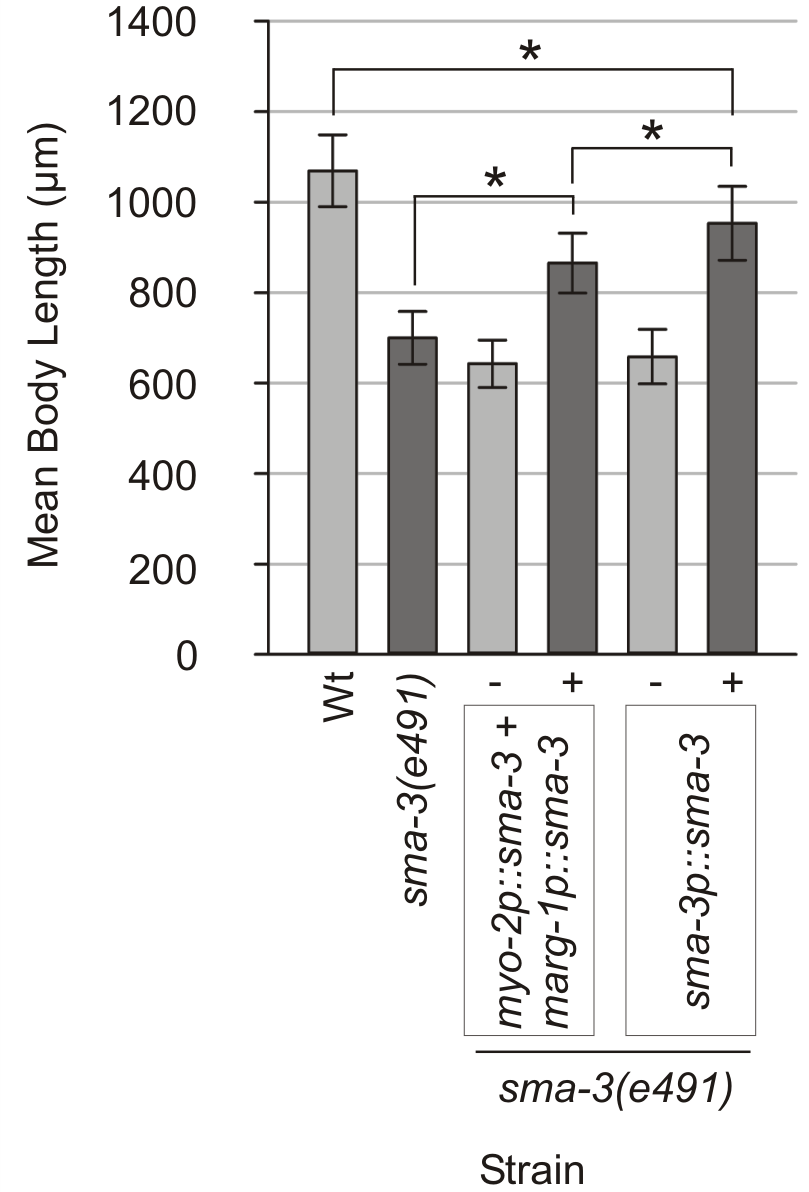

Supplement: Additional file 6: Figure S3. — Graphs of Pharynx and Body Lengths of Various Strains in the Absence of Anesthetic. Mean body length measurements ± standard deviation (measured under a dissecting microscope in the absence of anesthetic) of Wild type (Wt) N2, sma-3(e491) and sma-3(e491) animals from various sma-3 minigene rescue experiments. Vertical labels indicate tissue specific promoter-sma-3 minigene fusion rescue constructs in each strain. In each case, we measured animals carrying the transgenic array (+) and siblings that lacked the array (−), as before. Complete data for all lines is provided in Additional file 5. All transgenic animal means (+) were statistically significantly different from non-transgenic sibling means (−) (p<0.001). * denotes significant differences of p<0.001. All other differences in body lengths between strains not directly indicated on the graphs are significant (p<0.05). [file 12861_2014_43_MOESM6_ESM.tiff]

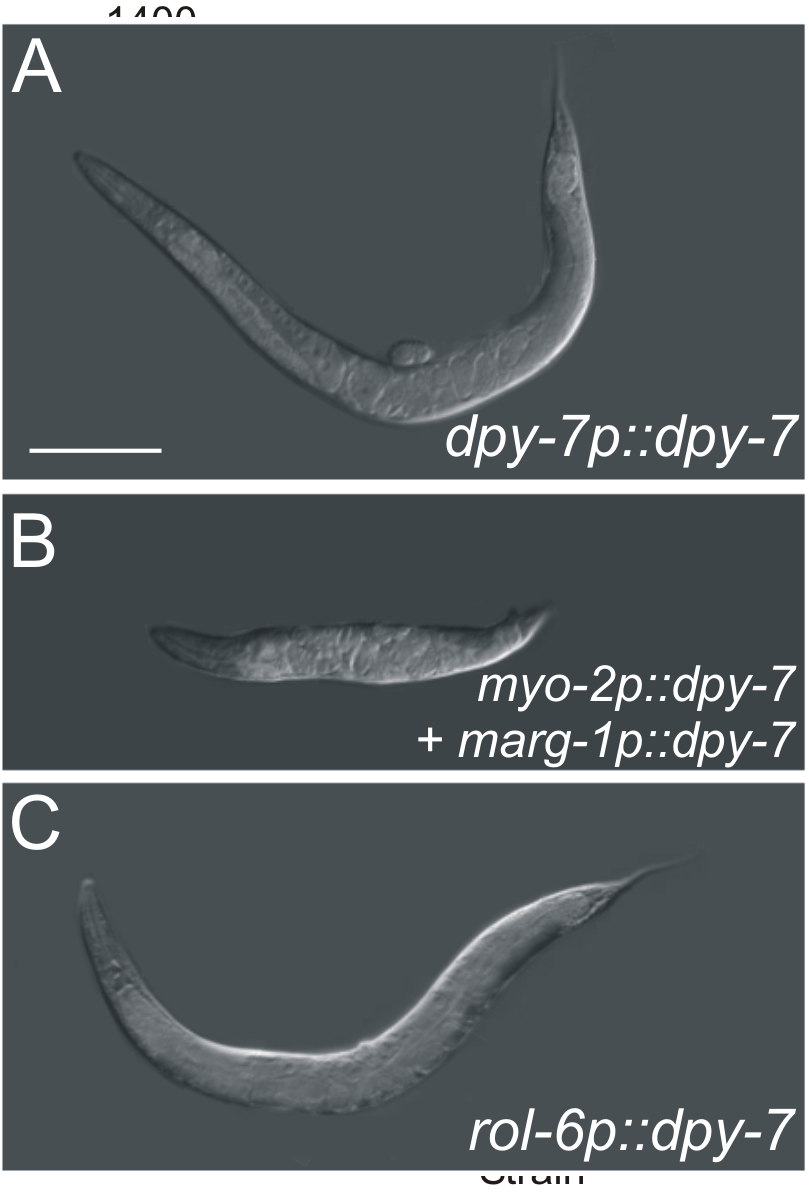

Supplement: Additional file 7: Figure S4. — Images of dpy-7 Rescue Experiment Phenotypes. Rescue of dpy-7 mutants by different transgenes. (A) Rescue of dpy-7 by a dpy-7p::dpy-7 transgene. (B) Expression of dpy-7 under the control of the pharyngeal promoters myo-2 and marg-1 does not rescue the dpy-7 phenotype. (C) Rescue of dpy-7 by a rol-6p::dpy-7 transgene. Complete data is provided in Table 1. Scale bar is 150 μm. [file 12861_2014_43_MOESM7_ESM.tiff]

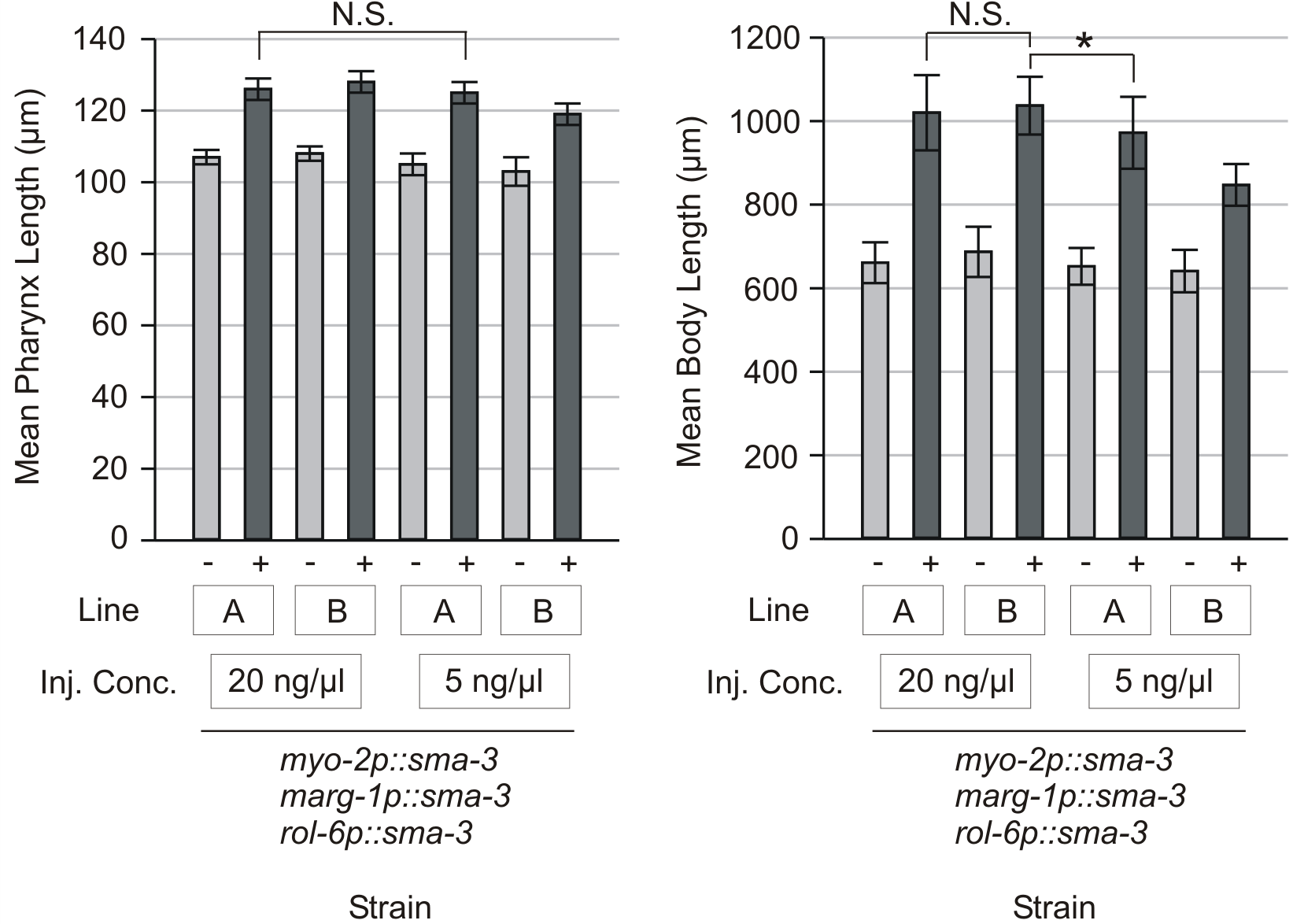

Supplement: Additional file 8: Figure S5. — Reduction of transgene dose has only minor effects on sma-3 rescue. Measurements are means ± standard deviation. Transgenic lines were established using injection mixes containing myo-2p::sma-3, marg-1p::sma-3 and rol-6p::sma-3, each at a concentration of either 20 ng/μL or 5 ng/μL, as indicated. A and B indicate independently generated transgenic lines. All transgenic animal means (+) were statistically significantly different from non-transgenic sibling means (−) (p<0.001). All differences in pharynx length not directly indicated on the graph are significant (p<0.001), except where indicated by N.S. * denotes significant differences of p<0.05. All other differences in body length not directly indicated on the graph are significant (p<0.05) except where indicated by N.S. Complete data for multiple lines is provided in Additional file 5. [file 12861_2014_43_MOESM8_ESM.tiff]
